# The threat sensitivity scale: A brief self-report measure of dispositional sensitivity toward perceiving threats to physical harm

**DOI:** 10.1038/s41598-024-61476-7

**Published:** 2024-05-17

**Authors:** David S. March, Connor Hasty, Vincenzo Olivett

**Affiliations:** Florida State University, 1107 W. Call St., Tallahassee, FL 32304 USA

**Keywords:** Threat, Sensitivity/bias, Perception, Danger, Physical harm, Scale, Human behaviour, Social evolution

## Abstract

The possibility of experiencing physical harm caused by an object, animal, or person is an omnipresent risk in almost any situation. People show variability in their in the propensity to perceive the possibility of harm from any ostensibly innocuous object or situation—a so-called threat bias. Despite the important psychological and societal consequences resulting from individual differences in physical threat bias, there does not currently exist an easily administered means to capture this disposition. We therefore endeavored to create a brief reliable self-report index of threat sensitivity for use by the many fields interested in the role of threat processing. We present here a physical threat sensitivity scale (TSS) that captures the dispositional tendency to perceive the possibility of physical harm in ostensibly innocuous situations or objects. We detail the development and validation of the TSS as a reliable index of individual threat bias (Studies 1a and 1b) and provide strong convergent evidence of the relationship between TS and both relevant individual differences (Study 2) and behavioral and perceptual indicates of threat bias (Study 3 and Study 4).

## Introduction

The possibility of experiencing physical harm caused by an object, animal, or person is an omnipresent risk in almost any situation. Walking down a city street exposes one to a variety of physical threats from, for example, a stranger who may have a gun, a dog who may be angry or rabid, or a driver of an automobile who may be distracted. Hiking in the woods exposes one to the threat of, for example, a snake that may be venomous, a wild predaceous animal, or a height of which one may fall. As an adaptive response to endemic risk, humans have evolved the ability to efficiently process threat. Relative to non-threatening cues in our environment, threat cues are prioritized via both cognitive and sensory processes^1–4^. Such preferential threat processing–dubbed a *threat superiority*^5–7^–facilitates humans' safe navigation of a complex and potentially dangerous social world. However, none of the above objects, people, or situations are inherently threatening. Most strangers are harmless, most stray dogs are more scared of you than you need be of them, most drivers are paying attention, only 10% of the world’s snakes are venomous, encountering a wild predator is an exceptionally uncommon experience, and being careful around ledges is usually enough to avoid an injurious fall.

Regardless of these base rates, people show variability in their in the propensity to perceive the *possibility* of harm in any ostensibly innocuous object or situation—a so-called *threat bias*. Underpinning the idea of a threat bias is the theory that “some people seem to go through life more cognizant of threats than others”^8^. Here people are more or less *sensitive* to the *possibility* of harm, even in situations where no harm is innate. Threat bias is often exemplified as attentional bias in, for example, dot probe and eye movement tasks^9,10^ and been linked to significant social phenomena, including, for example, a propensity towards certain political orientations and beliefs^11^, the likelihood of forming prejudices toward certain racial and ethnic groups^12^, and as central to the development of anxiety disorders in children and adults^13,14^. Indeed, contemporary research suggests that the threat of physical harm plays a central role in many phenomena such as prejudice^15–18^, attitudes^19,20^, emotion^21^, morality^22^, responses to COVID-19^23,24^, phobias and psychopathology^25,26^, and clinical work exploring antecedents to suicide^27,28^. Elucidating the role of threat has provided tremendous insight into a vast range of psychological phenomena.

Despite the important psychological and societal consequences resulting from individual differences in physical threat bias, there does not currently exist an easily administered means to capture this disposition. We therefore endeavored to create a brief reliable self-report index of threat sensitivity for use by the many fields interested in the role of threat processing. We present here a physical threat sensitivity scale (TSS) that captures the dispositional tendency to perceive the possibility of physical harm in ostensibly innocuous situations or objects. The scale is projective in nature—participants rate their perceived likelihood of being physically harmed by a range of objects or situations, none of which are inherently physically threatening. In other words, although the objects and situations lack any native threat-relevance, relatively more threat sensitive people may perceive that those situations present commensurately more potential for physical harm. In the following sections, we detail the development and validation of the TSS as a reliable index of individual threat bias (Studies 1a and 1b) and provide strong convergent evidence of the relationship between TS and both relevant individual differences (Study 2; e.g., Behavioral Inhabitation/Activation, Belief in a Dangerous World, Morbid Curiosity) and behavioral and perceptual indicates of threat bias (Study 3 and Study 4).

## Current measures of “threat sensitivity”

### The dot probe paradigm

The dot probe task has been used extensively to assess attentional bias toward threat^29^, and has become a gold-standard measure of threat bias^10,30^. In a threat-bias dot probe task, participants see cues, both threatening (e.g., angry faces, threat words or objects) and of an opposing category, often neutral. After a variable delay, the cues are replaced by a target of which the participant is tasked with categorizing the location. The target appears either in the same location as the threat cue (i.e., congruent trials) or the neutral cue (i.e., incongruent trials), and the difference between these two trial types is interpreted as an index of threat bias^31^. Unfortunately, the dot probe task is both difficult to administer (i.e., requires specialized software and analyses techniques) and more specifically an index of attentional bias assumed to be a manifestation of an underlying threat-driven vigilance, not a direct measure of threat sensitivity. In other words, the dot-probe task is an indirect index of attentional bias to threat; yet even that position has come under scrutiny as such tasks have been found to have poor test–retest reliability and low internal reliability and many studies fail to find a threat bias in both non-clinical and clinically anxious populations^32^. Furthermore, the traditional dot probe task conflates vigilance driven orienting and difficulty of disengagement. Specifically, a threat bias could be found as a consequence of both attentional vigilance for threat and/or difficulty to disengage attention from threat, with some work arguing that threat bias in dot probe tasks is likely driven more by the latter^33,34^. The dot probe is therefore best considered a measure of post-encounter vigilance and accordingly is a poor index of threat sensitivity.

### The Belief in a Dangerous World scale

Although no direct measure of threat sensitivity exists, there are several measures that have been used to approximate individual threat bias. The most prevalent of these—perhaps due to the ease of administering a self-report measure—is the Belief in a Dangerous World (BDW) scale^35^. The BDW scale was originally designed to explore the development of right-wing authoritarianism, specifically how the socialization of children reflected individual differences in the perception of threats connoted by people deemed dangerous or violent and belief that society is degenerating into chaos. Items indexed how much, for example, one believes that, “it seems that every year there are fewer and fewer truly respectable people, and more and more persons with no morals at all who threaten everyone else”, “there are many dangerous people in our society who will attack someone out of pure meanness, for no reason at all”, and “every day, as our society becomes more lawless, a person’s chances of being robbed, assaulted, and even murdered go up and up”. Influential work has shown that BDW is an individual difference predictor of phenomena as varied as prejudice towards groups stereotypically associated with safety threat^36,37^, conservative political beliefs^38^, and the development of certain moral foundations^39^. When used to assess belief in social threat connoted by specific others, the BDW is an appropriate individual difference predictor of myriad outcomes.

Unfortunately, likely resulting from the absence of a validated and easily administered threat sensitivity index, the BDW has in many cases been taken beyond its original scope to index a more general threat sensitivity. For example, researchers have used the BDW to explore whether people who exhibit an increased focus on threats to personal safety also evince increased bias towards threat-stereotyped groups^36^. Some authors refer to the BDW as a general measure of “safety threat” and “threat sensitivity” and reference work focused on basic threat management systems meant to avoid all sources of threat^40,41^. Indeed, some have liberally operationalize the BDW as “a scale to capture individual differences in chronic beliefs about the safety-relevant dangers present in the world” (p. 586). Likewise, in work exploring the role of dispositional and situational threat sensitivity in moral judgments, researchers used the BDW scale as a measure of “threat-sensitivity, broadly construed”^39^ (p. 386). This conceptual looseness conflates general threat sensitivity with more specific concerns about threats from deviant others of which the BDW was designed to index. The BDW is therefore best considered a crude or approximate measure of a specific threat sensitivity indexing one’s belief in the breakdown of social order. Consequently, BDW should be correlated with but distinct from a more general threat sensitivity scale.

### Behavioral responses measured via stabilometric force platform

When exposed to a threat, people both automatically lean away from the threat and engage in enhanced defensive freezing^42^. These behaviors can be captured in the lab by indexing spontaneous postural sway. When standing, people automatically and naturally engage many of the muscles in their lower extremities. Resulting from these small excursions, center of pressure is constantly shifting manifesting as visually unnoticeable postural movements known as spontaneous postural sway. In the lab, postural sway is indexed by having people stand on a stabilometric force platform that dynamically traces the person’s center of pressure (COP) on both X- (i.e., mediolateral, side-to-side) and Y-planes (i.e., anterior–posterior, front-to-back). Evidencing defensive behavioral responses, people freeze more when exposed to human and animal threats^43,44^ and when anticipating physical harm^45,46^ and lean more rearward in response to threat stimuli^47^. Unfortunately, capturing COP requires costly specialized often custom-built hardware and software, ample lab space, and in-person data collection. Furthermore, COP measures the *magnitude* of the threat response, not sensitivity to the possibility of threat. That is, whether measured as freeze or anterior–posterior movement, COP indexes the degree to which one behaviorally *responds* to the presence of an objective threat. Threat sensitivity, though, is a propensity to perceive the possibility of harm in stimuli, objects, or situations, regardless of whether they actually represent an objective threat. Although sensitivity may be correlated with (and perhaps potentiate) a response, they are not the same thing. Although posturography may be ideal for evaluating certain specific fears such as fear of heights, COP is a poor index of threat sensitivity.

## Current Work: Development of the threat sensitivity scale

That there exists no easy to administer and validated index of threat sensitivity is a critical limitation to research on the implications of individual differences in threat bias. The current work provides this measure by creating a short (5-item) scale that can be quickly and easily administered in any setting to assess idiosyncratic threat sensitivity. This index reflects the view that threat bias and sensitivity reflect the dispositional tendency to perceive the possibility of physical harm in situations or objects both innocuous and not. The primary goal of Study 1 was to explore the factor structure of a set of many items that were intended to assess general threat sensitivity with the goal of developing a 5-item single-factor scale. We used exploratory factor analysis to drop items with low factor loadings to generate a shortened list of items with a coherent factor structure. Study 2 provides convergence with relevant individual differences, and Studies 3 and 4 provide convergence with a perceptual index of threat bias and an instantiation of the behavioral defensive response, respectively.

All experimental protocols were approved by the institutional review board at Florida State University. All methods were carried out in accordance with relevant guidelines and regulations. Informed consent was obtained from all participants prior to participation.

## Study 1: Item development and reliability

We developed a set of items encompassing a range of objects, situations, or others who lack innate threat-relevance but could connote threat under certain circumstances. This generated an initial list of nineteen items (See Table 1 for all items). For systematic scale development, in Study 1a we performed factor analyses to remove items that did not load well into a single factor, removed items that were redundant, vague, ambiguous, or could activate phobias or biases. In Study 1b, we assess this factor structure and reliability of the short scale in a larger, more demographically balanced independent sample.

**Table 1:** Threat sensitivity scale, first round including all items.

Item	Factor loading	Item	Factor loading
1. Unlocked door	0.50708	11. Swimming in the ocean	0.59560
2. Unlocked window	0.52901	12.* Using a knife to cut food*	*0.48667*
3.* Drunk person*	*0.42907*	13. Walking in a dark alley	0.61871
4. Road trip	0.50025	14.* Walking in a field*	*0.46224*
5. Stranger	0.62080	15.* Hitchhiking*	*0.39789*
6. Camping in the woods	0.71116	16.* An unknown dog*	*0.46965*
7. Old wooden bridge	0.62334	17.* A police officer*	*0.45110*
8. Spider	0.50662	18. Flying in an airplane	0.54251
9. Edge of a mountain	0.64240	19. Riding in an elevator	0.54482
10. Going on a ride at the fair	0.51876		

Items in *italics* are removed at this stage.

### Study 1a

We collected data from 206 participants convenience sampled from the undergraduate population at a large southeastern American university. Participants received partial course credit. One-hundred seventy-five participants identified as women, 30 as men, and 1 did not report. Their mean age was 19.

Participants were told that we were interested in their perception of objects and situations. They were prompted to “*Imagine you come across the following objects, people, or are undertaking the following situations. Please indicate how likely it is that you would be physically harmed by each object or in each situation*.” Responses were rendered on a 1–7 scale (*1* = *Unlikely*; *4* = *Neither likely or unlikely*; *7* = *Likely*). Items were presented in random order.

In selecting items, we aimed to create a single-factor, 5-item, brief questionnaire that could be easily administered by future researchers across domains with different technological resources and capabilities. To that end, we aimed for a minimal number of items while maintaining proper psychometric properties. We also considered theoretical issues pertaining to the content of those items to ensure none were more inherently threatening than others. First, we conducted principal axis factoring to narrow items to only those that loaded onto the primary factor above our a priori determined minimum threshold (0.5) and re-ran the factor analysis with the remaining items until we reached the final 5 items. As our goal was to retain a single factor, varimax rotation was used to encourage items to load on only a single factor.

The first round of factor analysis did not specify a number of factors which allowed each item to load on a number of factors equal to the number of items. This allowed us to identify the items that most strongly loaded onto the primary factor. See Table 1 for the factor loadings of each item in round 1.

In this first round, the primary factor explained 74% of variance with an eigenvalue of 5.65. No other factor loaded above an eigenvalue of 1.25, supporting our theory that the threat sensitivity scale can be represented by a single primary factor. In the second round, we ran the identical model while specifying a single factor. Of the original 19 items, we cut 6 items that did not load above 0.5. After the second round, we cut 3 items that did not load above 0.5. See Table 2 for the factor loadings of each item in round 2.

**Table 2:** Threat sensitivity scale, second round.

Item	Factor loading	Item	Factor loading
1. Unlocked door	0.51274	9. Edge of a mountain	0.66783
2. Unlocked window	0.54380	10.* Going on a ride at the fair*	*0.48456*
4.* Road trip*	*0.47831*	11. Swimming in the ocean	0.60527
5. Stranger	0.59161	13. Walking in a dark alley	0.60828
6. Camping in the woods	0.71286	18. Flying in an airplane	0.57001
7. Old wooden bridge	0.62732	19. Riding in an elevator	0.55596
8.* Spider*	*0.48564*		

Items in *italics* are removed at this stage.

All of the remaining 10 items loaded at greater than 0.5 in the third round. From the remaining 10, we removed items that were deemed redundant (e.g., we retained “an unlocked door” while removing “an unlocked window”), where the source of threat was ambiguous (i.e., “riding in an elevator”, “flying in an airplane”), that may be highly influenced by attitudes about class and race (i.e., “walking in a dark alley”), that may only be applicable to people living in Western societies and not those in Majority World contexts (i.e., many people have never flown in an airplane or rode in an elevator), and strove to balance social and non-social sources. See Table 3 for the final 5 item set and their factor loadings from the fourth round of factor analyses This scale (*M* = 4.12) demonstrated good internal reliability (α = 0.77).

**Table 3:** Final threat sensitivity scale.

Item	Factor loading
1. Unlocked door	0.53527
5. Stranger	0.55729
6. Camping in the woods	0.68750
7. Old wooden bridge	0.69505
9. Edge of a mountain	0.71617

Given the young and female-biased sample of this first study, we aimed to replicate this factor structure in a larger, more demographically balanced sample.

### Study 1b

We collected data from 304 participants via Amazon’s Mechanical Turk who passed attention checks and had complete data. One-hundred forty participants identified as women, 163 as men, and 1 did not report. Their mean age was 42.5.

**Figure 1:**
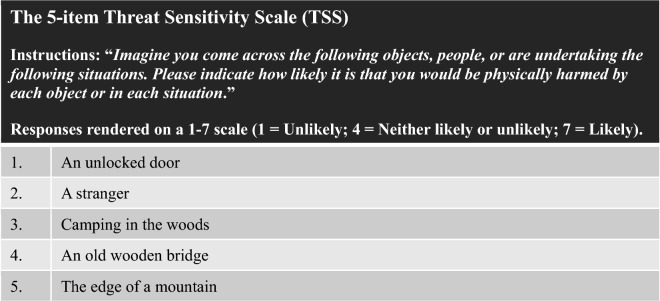
The full final threat sensitivity scale.

We replicated the factor structure and reliability of the single-factor 5-item TSS via confirmatory factor analysis in SAS. The 5-item scale (*M* = 3.43) demonstrated good reliability (α = 0.88) without being redundant. See Table 4 for items and factor loadings. Model fit indices suggest excellent fit with an SRMR = 0.029 (less than 0.08 is considered well-fitting^48^), GFI = 0.965 (greater than 0.95 is considered well-fitting^49^), CFI = 0.974 (greater than 0.96^48^ or 0.90^49^ is considered well-fitting), and NFI = 0.969 (also called the TLI, the NFI is preferable for smaller samples; greater than 0.90^49^ or 0.95^50^ is considered well-fitting). Together Studies 1a and 1b demonstrate that our threat sensitivity scale (TSS) is well-formed and internally reliable. For the ease of future researchers, the full scale and instructions can be found in Fig. 1.

**Table 4:** Threat sensitivity scale, study 1b.

Item	Factor loading
1. Unlocked door	0.61585
5. Stranger	0.79490
6. Camping in the woods	0.89202
7. Old wooden bridge	0.83127
9. Edge of a mountain	0.76140

Next, we aimed to provide evidence that the TSS captures a unique construct related to but not identical to other measures theoretically convergent or divergent to threat sensitivity.

## Study 2: Convergence and divergence with other self-report measures

The purpose of Study 2 was to assess the convergent and divergent validity of the TSS among several diverse samples. In considering the convergence of our novel scale, we identified existing measures that in some way relate to the perception of immediate threat. To that end, we identified the above-reviewed BDW scale, the Disgust Propensity and Sensitivity Scale-Revised (DPSS-R^51^), the scale measuring dispositional Behavioral Inhibition/Activation System sensitivities (BIS/BAS^52^), the Curiosity about Morbid Events (CAME) scale^53^, and our Modern Morbid Curiosity Scale^54^ as concepts that should converge or diverge with threat sensitivity. We also identified the Big Five personality Traits via the Ten-Item Personality Inventory^55^ concept of Emotional Stability (i.e., the inverse of neuroticism) as something that should diverge with threat sensitivity.

### Belief in Dangerous World (BDW) scale

Participants responded to the 13-item BDW scale^35^ by indicating how much they believed each statement (e.g., *There are many dangerous people in our society who will attack someone out of pure meanness, for no reason at all*) was true or false on a 7-point scale (1 = *Not true at all* to 7 = *Very* t*rue*). To the degree that higher TS and BDW reflect a similar but not redundant disposition, the TSS should positively relate to the BDW scale.

### Disgust propensity and sensitivity scale-revised (DPSS-R)

Participants responded to the 12 item DPSS-R^51^ by indicating how often each statement is true for them on 5-point scale (*Never, Rarely, Sometimes, Often, Always)*, such as *I avoid disgusting things.* To the degree that higher TS and disgust sensitivity develop from a similar but not redundant system, the TSS should positively relate to the DPSS-R.

### Behavioral inhibition/activation system (BIS/BAS) scale

Participants responded to the 20-item BIS/BAS scale^52^ which contains subscales indexing two dimensions of personality reflecting (1) an aversive motivation system (BIS) which regulates aversive motives toward avoiding something unpleasant and (2) an appetitive (approach) motivational system (BAS) which regulates appetitive motives toward attaining something desired. The BAS subscale is further divided into those indexing a Drive subscale pertaining to goal pursuit, a Fun Seeking subscale pertaining to spur of the moment action, and a Reward subscale indexing responsiveness to reward. Our focus was on the BIS and, its complement, the Reward subscale (though we report all subscales). Those higher in TS may both evince higher sensitivity to punishment via BIS and greater drive for reward (i.e., the inverse of punishment). Consequently, the TSS should positively relate to both BIS and the Reward subscale and have no relation to the Fun Seeking and Drive Subscales.

### Curiosity about morbid events scale (CAMES)

Participants responded to the 28-item CAMES^53^ by indicating true/false to items that assess an interest in watching violence and death, such as *I enjoy being mildly frightened by horror movies* and *I would not want to look at a dead person*. To the degree that higher TS discourages engaging in fear-inducing behaviors, the TSS should negatively relate to the CAMES.

### Modern morbid curiosity scale (MMCS)

Participants responded to the 10-item MMCS^54^ which assesses how much attention or interest they would exhibit if they were to see each several objects or situations (e.g., *a car accident, screaming in the distance*) on a 5-point scale (*I would pay no attention, I would pay a little attention, I would pay an average amount of attention, I would pay a fair amount of attention, I would pay a lot of attention*). The MMCS has assesses people’s interests in gathering information relevant to potentially but not immediately threat-relevant situations or objects of crime and human punishment. MMC is operationalized as an “attentional approach disposition toward certain ambiguous stimuli with the integrated goals of (1) gathering immediate and future relevant information and (2) determining the nature of the stimuli. In other words, survival relevant information gleaned from certain negative stimuli engenders a temporary attentional approach motivation with the intention of determining whether avoidance is necessary”^54^. Consequently, to the degree that TS encourages seeking information relevant to reducing one’s exposure to threatening objects or situations, the TSS should positively relate to the MMCS.

### Ten-item personality inventory (TIPI)

Participants responded to a10-item scale^55^ indexing Extraversion, Agreeableness, Conscientiousness, Emotional Stability, and Openness to Experience by thinking about how much each item described them on a 7-point scale from 1 = *disagree strongly*, 4 = *neither agree nor disagree*, to 7 = *agree strongly*. Our focus was specifically on the Emotional Stability subscale, which indexes calmness and stability, and is the opposite of neuroticism (i.e., the dispositional tendency to experience anxiety and fear in everyday social situations). To the degree that higher TS is related to (and perhaps underlies) a neurotic disposition, TSS should negatively relate to the Emotional Stability (ES) subscale (i.e., higher TS corresponds to lower ES [higher neuroticism]).

### Results and discussion

TSS (*M* = 3.65) data were recorded from a total of four-thousand nine-hundred and fifty-five participants (*M*_age_ = 35.7; 1,915 male, 3,009 female, 33 other or unreported; 3,566 White, 432 Black, 562 Asian, 397 other or unreported) across several unrelated studies. The TSS demonstrated good internal reliability (*α* = 0.82). Within each study, the TSS was included as part of the questionnaire portion. Given that convergence tests originate from unique sub-samples, sample size information for each test is provided with its associated test. For descriptive reasons, we tested the relationship between TSS and age and gender, respectively. A linear regression revealed a negative relationship between Age and TSS, *b* =  − 0.004, *t*(4954) =  − 3.82, *p* < .001. ANOVA revealed that women (*M*_women_ = 3.83) exhibited higher TSS than did men (*M*_men_ = 3.38), *t*(4922) = 12.38, *p* < .001. To provide evidence for convergent and discriminant validity, we present in Tables 5 and 6 correlations between the TSS and each of the above-listed scales.

**Table 5:** Correlations between TSS and primary measures of convergence and divergence.

Scale	Mean (SD)	N	*r*	*p*
BDW	4.2 (.99)	725	0.18	< .001
DPSS-R	2.66 (.58)	208	0.29	< .001
BIS	1.82 (.43)	195	0.32	< .001
BAS-R	1.58 (.42)	195	0.14	.056
BAS-D	2.27 (.57)	195	0.05	.44
BAS-FS	2.05 (.50)	195	− 0.05	.45
MMCS	3.46 (.63)	434	0.23	< .001
CAMES	8.15 (3.34)	208	− 0.18	.008

BDW, Belief in Dangerous World; DPSS-R, Disgust Propensity and Sensitivity Scale-Revised; BIS, Behavioral Inhibition System; BAS-R, Behavioral Activation System-Reward; BAS-D, Behavioral Activation System-Drive; BAS-FS, Behavioral Activation System-Fun Seeking; MMCS, Morbid Curiosity; CAMES, Curiosity about Morbid Events Scale.

**Table 6:** Correlations between TSS and the big-five personality domains measured by the TIPI.

Scale	Mean (SD)	N	*r*	*p*
TIPI-ES	3.94 (1.57)	208	− 0.26	< .001
TIPI-A	4.71 (1.15)	208	− 0.007	.92
TIPI-C	5.47 (1.16)	208	0.05	.44
TIPI-E	4.25 (1.51)	208	− 0.08	.23
TIPI-OE	5.32 (1.08)	208	− 0.03	.64

ES, Emotional Stability; A, Agreeableness; C, Consciousness; E, Extraversion; OE, Openness to Experience.

The BDW scale correlated moderately with the TSS, as expected, but not excessively. Consistent with the idea that the BDW scale measures a unique and perhaps more narrow consequence of a general threat sensitivity, the BDW was positively related to TS. This confirms our earlier supposition that the BDW scale is not a measure of general threat sensitivity and should not be used as such.

A complementary pattern emerges for other conceptually related constructs such as DPS, MMC, and CAME. Here we see that TS is positively and strongly related to disgust sensitivity, but again not excessively so. This raises the empirical question of whether threat and disgust sensitives are manifestations of the same underlying system moderated by other individual differences or are orthogonally manifested via unique systems that result in moderately overlapping dispositions. The relationship with the MMCS supports our operationalization of morbid curiosity as an adaptive (i.e., fitness-enhancing) mechanism in response to certain survival relevant stimuli reflecting a brief state of motivational uncertainty (i.e., neither approach nor avoid) as additional information is gathered and a resolution reached. As the morbidly curious stimuli may contain survival relevant information, it follows that people higher in TS should be more motivated to gather such information. That information can then be incorporated into one’s model of the world and put to use in future attempts to avoid or address harm. This disposition is mirrored in the negative relationship between the TSS and the CAMES. Recall that the CAMES indexes a desire to engage in potentially frightening or scary experiences, hence people higher in TS should be less CAME. That is precisely what we see in the current data where those higher in TS exhibit less CAME. This implies that people higher in TS may also less enjoy fear-inducing experiences, even those done safely like watching scary movies.

The correlation between the TSS and BIS was modest, and in the direction we expected. The positive but not overlapping correlations indicates that TS may partially reflect an aversive disposition among those with relatively more active dispositional BI. Alternatively, the motive to avoid unpleasant events or occurrences may be in part due to a generally higher expectation that physical harm is a possible outcome. That is, those higher in TS who perceive a higher likelihood of harm in generic contexts and situations may be more disposed to avoid such contexts (i.e., be higher in BIS). Complementarily, people who perceive a higher likelihood of threat occurring may be more focused on obtaining safety, which here manifests as a marginally significant relationship with a Reward seeking disposition focused on nonpunishment. The lack of a relationship between the TSS and Fun Seeking or Drive confirms the original authors argument that the BAS subscales index different aspects of incentive sensitivity, with TS seemingly only relating to a safety-seeking disposition.

As can be seen in Table 6, the TSS only correlated with the Emotional Stability personality domain as the TSS was negatively related to ES. Here people higher in TS are experiencing less emotional balance and may experience more negative emotions. The negative relationship suggest that some degree of neuroticism is related to or driven by a general TS. Increased TS may give rise to some of the underlying causes of neuroticism that manifest as worry about one’s safety, accentuated stress, more frequently experience of negative emotions, and increased generalized anxiety. TS did not correlate with any other personality domain, confirming our position that TS is a unique disposition related to but not the same as neuroticism and not related to or reflective of more general personality traits.

## Study 3: Convergence with a measure of threat projection

**Figure 2:**
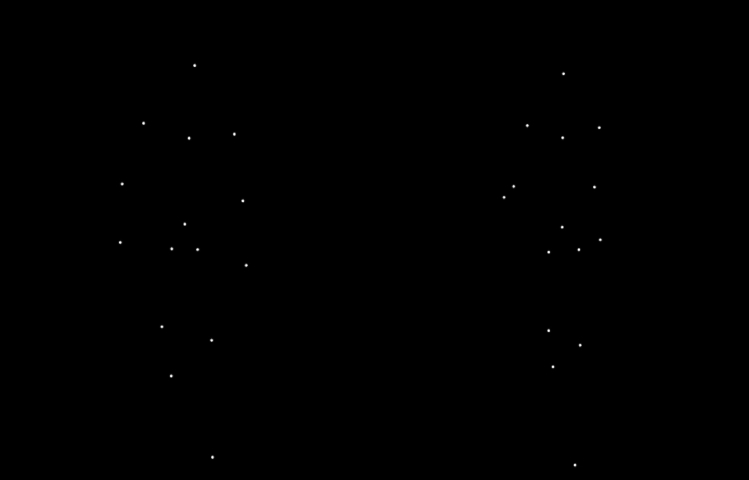
Images of point-light walkers.

Study 3 used ambiguous human targets in the form of point-light-walkers (PLWs) to explore whether people higher in TS also project more threat-capabilities onto the PLWs. A PLW is a collection of animated white dots depicting a walking human (see Fig. 2 for examples). Individual PLWs can vary along several dimensions, including size, speed, and direction of movement. Participants are typically told that PLW animations are constructed by tracking the actual movement of real people walking, when in reality they are artificially constructed. So, while individual PLWs ostensibly represent real humans who could perceivably vary in the mind of the perceiver in the extent to which they pose physical threats, they in fact do not. In other words, PLWs entail no inherent threat relevance but that projected onto them by the perceiver. In Study 3, we expected that people higher in TS would show a commensurately increased perception of the threat-potential of the PLWs. In other words, increased physical threat perceptions of PLWs should correspond with a generalized sensitivity to projecting physical threat onto other humans.

### Method

We collected data from 150 participants via Amazon’s Mechanical Turk who passed attention checks and had complete data (83 women, 65 men, 2 other or not reported; M_age_ = 47.28, SD_age_ = 14.94). Participants were told that we had captured movements of humans walking using light-points, and we were interested in their perceptions of these humans. Then, participants viewed one PLW at a time for as long as they wished before immediately reporting their perceptions of that PLW. Participants rated each PLW on three items assessing perceived physical threat (*How potentially dangerous do you think this person is to you?* [1 = Not dangerous at all, 7 = Extremely dangerous]; *I believe if I came into contact with this person I would be harmed*. [1 = Strongly disagree, 7 = Strongly agree]; *How threatening is this person?* [1 = Not threatening, 7 = Extremely threatening]). In total, each participant rated four PLWs varying slightly in size and distance from viewer. All PLWs were walking towards the viewer and dynamically became larger as they approached. After rating PLWs, participants completed the TSS (*M* = 3.60, α = 0.82) and a demographic questionnaire.

### Results and discussion

**Figure 3:**
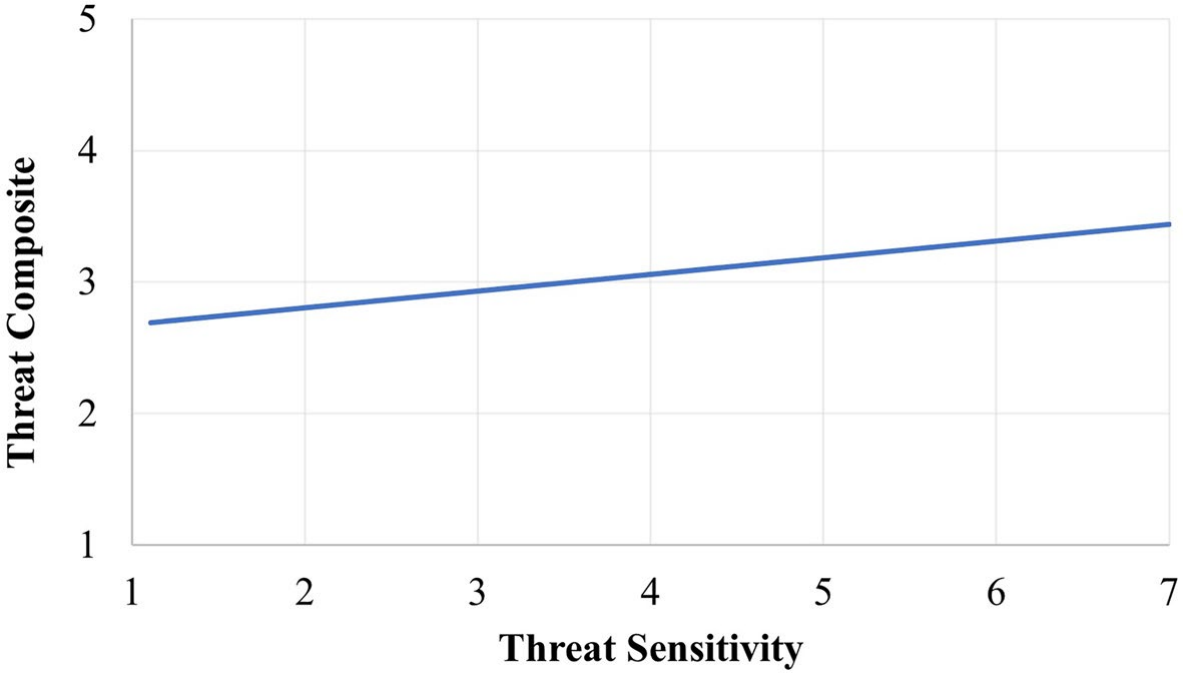
Relationship between threat sensitivity and threat composite ratings.

A composite PLW threat perception score was calculated for each subject by averaging the danger evaluations of the four PLWs. To assess the relationship between TS and the perception of the threat-potential of the PLWs, we regressed composite threat perception onto the TSS. This analysis revealed a positive relationship between TSS and PLW threat, *β* = 0.13, CI_95_ [0.04, 0.21], *SE* = 0.04, *t*(149) = 2.96, *p* = .004 (see Fig. 3). The observant reader may note that one item within the TSS is “A stranger” and ask whether this result is driven primarily by that single item within the TSS. To explore this consideration, we correlated each TSS item with the Threat Composite. Correlations were Unlocked Door *r* = 0.21, Stranger r = 0.17, Camping in the Woods *r* = 0.18, An Old Wooden Bridge *r* = 0.20 , Edge of a Mountain *r* = 0.15. Consequently, the effect found in Study 3 was not driven by a single item but by general threat sensitivity.

In Study 3, TSS is treated as an individual difference predictor of the degree to which people project the ability to inflict harm onto an ostensibly threat-neutral social target. Here we found that as people perceive the likelihood of experiencing harm in harm-agnostic situations via the TSS, they likewise perceive that a harm-neutral social other to be of greater potency (i.e., have an enhanced capability to inflict harm). This study indicates that threat sensitivity underlies—at least in part—a propensity toward increased perceptions of the harm capable of being inflicted by social (and perhaps non-social) others. This effect hints that threat sensitivity may also in part underlie other types of outcomes of which a component is the over- or mis-perception of harm potential. For example, individuals with obsessive compulsive disorder (OCD) and certain anxiety disorders often have fears that they will be harmed, often by an unknown or unnamable agent (5th ed.; DSM–5^56^). That TS is related to perceiving an ostensibly threat-neutral PLW as more dangerous implies that the same danger-projection may occur towards many classes of stimuli, and perhaps more diffusively as in the case of neuroticism and some cases of generalized anxiety disorders. The role of TS is psychopathology is a promising avenue for future research.

## Study 4: Convergence with an instantiation of the threat response

When encountering a threat, escape is always the preferential option. Yet sometimes escape is not an immediate option. In humans, information seeking in the form of visual and psychological attention is increased when the opportunity to escape is not an option^57^. Recent research has suggested that effect results via the activation of instrumental behaviors aimed at reducing the likelihood of experiencing harm. That is, people may seek out information in the presence of a threatening stimulus toward the goal of determining the best next course of action. In the threat processing literature, enhanced defensive freeze via a reduced bodily sway is a means of avoiding detection^42,43,45,46,58,59^. Yet escape necessarily entails gathering information (e.g., a route of escape), involving the dynamic shift of one’s attention within the environment. This is especially true if escape is not immediately available. Defensive freezing is assessed via whole-body postural sway (i.e., how much peoples’ body weight shifts while viewing stimuli) captured using a stabilometric force platform that continuously records a standing person’s center of pressure^44^. If in the presence of a threat people are actively scanning the scene to gather information, their body sway should be more pronounced than people in a more active freeze state. With regard to TS, those highest in TS may be the most motivated to avoid harm, and hence may either (a) exhibit enhanced freeze to avoid immediate detection or (b) exhibit enhanced sway as a means of finding an escape. For those lowest in TS, we would expect enhanced sway as their reduced TS is unlikely to evoke an increased freeze and instead encourage gathering the means toward making an active escape (if and when one becomes available).

In the current Study 4, participants stood on a force-platform and viewed threatening images while their sway was dynamically calculated across repeated trials. If sway decreases linearly with increased TS, we would expect a (a) linear trend whereby increased TS results in reduced sway. Alternatively, if those highest (and lowest) on TS are those more actively seeking a means of escape, we would expect a quadratic (u-shaped) trend where those at the extremes of TS are evincing more sway than are those closer to the mean.

### Method

We collected data from 201 undergraduate participants (131 women, 62 men, 8 other or not reported; M_age_ = 19.29, SD_age_ = 2.03) for participated or partial course credit at a large southeastern American university.

Participants undertook Study 4 in a private room equipped with a height-adjustable monitor, a computer, and a custom built 50.8 × 50.8 cm force platform. The platform was equipped with four load sensors (one at each corner) and sampled center-of-pressure (COP) at a rate of 83 Hz (one sample/12 ms). Prior to each session of data collection, the platform was tared to zero-zero and the monitor height was adjusted to approximately the participant’s eye level. Participants were instructed to remove their shoes and step onto the balance board after it has been calibrated (i.e., tared to zero-zero). They were then told to stand still on the platform with feet approximately 20 cm apart (on a sticker indicating foot placement) and to passively view a block of images. Participants were instructed to pay close attention because they would be asked to recall the images at a later time. Then, participants passively viewed 25 continuously presented threatening images (e.g., shark, snarling wolf, snakes, etc.; stimuli were previously piloted to ensure they were deemed physically threatening^5^) lasting 3 s each, after which they completed the TSS (*M* = 3.60, α = 0.78) and a demographic questionnaire.

### Results and discussion

Prior work shows that diminished sway in the anterior–posterior (AP; i.e., front to back) plane reflects threat induced motor freezing in humans^44,46^. We therefore focused analyses on AP sway. We quantified sway in the AP direction as mean standard deviations (AP-SD) of COP within each 3-s trial^44^.

**Figure 4:**
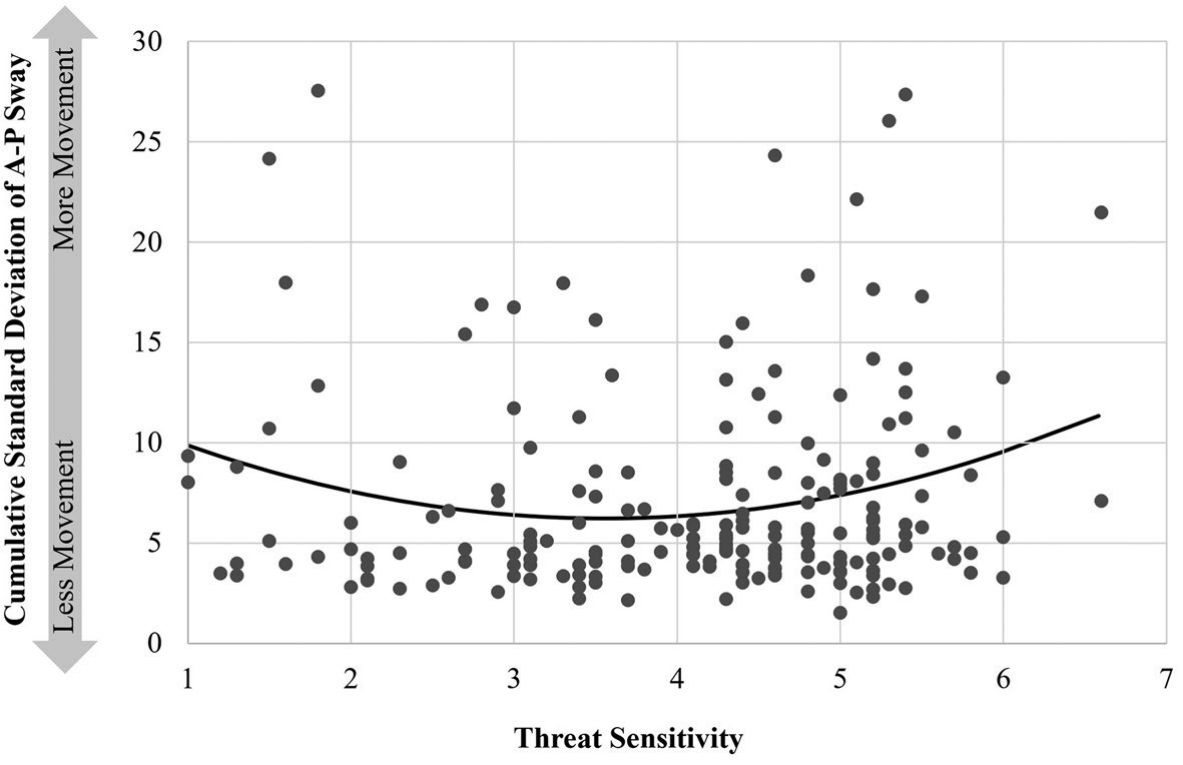
Quadratic relationship between threat sensitivity and sway.

The test the linear relationship between TSS and AP-SD, AP-SD was regressed onto TSS. This test revealed no linear relationship between AP-SD and TSS, *F*(1,200) = 0.54, *p* = .588. The test the quadratic relationship between TSS and AP-SD, AP-SD was regressed onto TSS and its quadratic term (TSS*TSS). This test revealed a quadratic relationship between AP-SD and TSS, *F*(2,199) = 7.04, *p* = .009, reflecting higher AP-SD among those relatively lower and higher than the mean of TSS (see Fig. 4).

In Study 4, TSS is treated as an individual difference predictor of the degree to which people freeze when exposed to objectively threatening stimuli. Here we found that people at the poles of TSS tend to sway more in the presence of threat than do people closer to the middle of the scale. This likely reflects that people at the low end of TS are actively scanning (relative to those in the middle), gathering information should an opportunity to escape present itself. People at the high end of TS, who one may expect to freeze the most, are likewise evincing increased information seeking tendencies relative to those in the middle, perhaps reflecting a relatively heightened motivation to escape. This seemingly non-intuitive finding highlights the unique insights that can be gleaned by using the threat sensitivity scale.

## General discussion

We propose the need for a general threat sensitivity scale by highlighting the many fields of psychology interested in dispositional threat sensitivity but also the lack of a valid measure of the construct. We detailed how we developed and validated the psychometric properties of an easily implemented self-report threat sensitivity scale. We described how we formed the initial scale and iterated via factor analysis to a succinct and highly internally reliable 5-item scale. We then confirmed the structure and reliably of the TSS in a large representative sample and presented data attesting to the convergent validity, discriminant validity, and divergent validity of the TSS with theoretically related and unrelated constructs. Lastly, we present two studies exhibiting that the TSS predicts perceptual and behavioral measures of threat. Specifically, Studies 3 and 4 exemplify both the construct validity of the TSS and its utility for broader psychological science via evidence that TS relates to threat-projection on ostensibly threat-neutral social objects and defensive responses in the presence of objectively threatening stimuli. Together these studies serve to make clear the validity and utility of the TSS as a measure of generalized threat bias.

The TSS indexes an individual’s tendency to perceive the possibility of harm (i.e., the threat potential) in ostensibly innocuous situations or objects. Critically, the scale is projective in nature—participants rate their perceived likelihood of being physically harmed by a range of objects or situations, none of which are inherently physically threatening. In other words, although the objects and situations lack any native threat-relevance, relatively more threat sensitive people may perceive that they present commensurately more potential for physical harm. Contemporary research suggests that the threat of physical harm plays a central role in many phenomena such as prejudice^15–18^, attitudes^19,20^, emotion^21^, morality^22^, responses to COVID-19^23,24^, phobias and psychopathology^25,26^, and clinical work exploring antecedents to suicide^27,28^. Elucidating the role of threat has provided tremendous insight into a vast range of psychological phenomena. Left untested by this work is the dispositional differences in individual threat sensitivity that may uniquely dispose certain people to evince threat-driven or threat-consistent responses. Indeed, a more threat sensitive person may be particularly prone to developing and evidencing prejudices underlaid by threat associations, for example a Black-threat association^16,60^. And, alternatively, a person who is relatively less threat sensitive may be more capable of or of acquiring the capability to inflict the personal self-harm necessary for suicidal acts^61^.

Furthermore, a more threat sensitive person may be more likely to develop certain phobias and psychopathologies that are underlaid by harm avoidance. Indeed, different objects and situations pose different types of “threat” (i.e., physical vs. contamination) and not all rise to the degree of phobic fear. Whereas the TSS focuses on threats to physical harm and may predispose one to developing certain phobias, other work focuses on threats to contamination and self-reported fear associated with specific phobias. For example, disgust sensitivity focuses on stimuli that typically evoke threats to contamination^51^, as when exposed to certain insects and blood, and is qualitatively unique to the TSS’s focus on stimuli and situations that evoke threat to physical harm^5,6^. And although the TSS focuses on one’s projective perception of the physical harm potential connoted by innocuous objects and situations, other work has focused on the amount of “fear” felt by individuals in response to potentially fear inducing objects or situations^62,63^. The most prevalent of these is the Fear Schedule Survey (FSS), a measure of specific phobia designed to index self-reported fear evoked by a wide range of potentially phobic situations and objects, such as crossing the street, people who seem insane, dental procedures, worms, enclosed places, etc. Whereas the FSS was designed for clinical use to index potential phobias by tapping self-reported fear (i.e., an emotion construct), the TSS instead taps granular differences in the amount of physical harm people project onto innocuous objects and situations. These scales each focus on a unique component of people’s idiosyncratic harm-avoidance processes and may when used in conjunction provide synergistic insights. These distinctions suggest the TSS will be useful both to evaluate individual differences in threat sensitivity, but also to distinguish between different fears and phobias. Indeed, being able to distinguish between perceptions of threat and disgust (and fear) is paramount to both researchers and clinicians striving toward building a comprehensive theory of human threat evaluation. These are only some examples of the myriad social outcomes that may vary as a function of threat sensitivity and the unique processing of threat^19,64^. By developing the TSS, we have provided a means to explore these outcomes using a reliable index of individual threat bias.

### Limitations and future directions

Notably, although our samples were generally representative of the US population, they were only from the US, which is a WEIRD population (Western, Educated, Industrialized, Rich, and Democratic)^65^. Although we strove to reduce the scale items only to those that would be familiar and relevant to people of many cultures and societies, future work would benefit from exploring the predictive validity of the TSS in non-western samples and from a variety of cultures. Although we expect that threat sensitivity as a construct is universal, its measurement may be influenced by important ecological factors that vary across nations and cultures.

Furthermore, although we strove to exhaustively test the convergence of the TSS with theoretically related constructs, perceptions, and behaviors, there are likely other measures that would be important to consider for further validation. For example, perhaps some tenets of terror management theory, for example, the terror produced by the inevitability of death while striving to live, is partially underlaid by increased threat sensitivity. Humans higher in TS may be more likely to ignore or avoid thinking about the inevitability of death (to the extent that death is threatening). That subconscious anxiety may be moderated by threat sensitivity. Also, it has recently been suggested that threat processing is related to political attitudes and beliefs, specifically that threat drives people to support more conservative versus liberal political policies. Yet recent replication failures^66^ have undermined this idea, which has left murky the relationship between threat and political outcomes. Some of our recent work has shown that heightened threat sensitivity on the TSS is associated with increased support for anti-democratic sentiments and increased support for partisan violence (e.g., support for anti-democratic ingroup politicians and policies and support for violent ingroup partisans). This pattern held even when controlling for political ideology and extremity. Meaning, regardless of political orientation, people who are more threat sensitive are likewise more likely to express support for anti-democratic norms and political violence. Some of our and other’s future work can employ the TSS to examine to what degree threat sensitivity underlies many political outcomes.

One last potential limitation is the scale’s narrow-scope, consisting only of five items. Although we offer compelling evidence supporting the scale’s validity, concerns may persist regarding the adequacy of the limited item count in capturing the multifaceted construct of threat. We designed the scale to capture our definition of threat sensitivity as reflecting a dispositional tendency to perceive a higher likelihood of being harmed in and by many ostensibly innocuous situations and objects. Hence, we whittled the original several dozen items down to 5 that well-captured the construct and showed excellent internal and external validity. To that end, we removed items from a remaining 10-items that loaded above 0.5 in Step 3 to increase the utility of the scale to a wide audience and encourage generalizability. We argue that having items more likely to tap the experiences of WEIRD individuals, racial or ethnic biases, or phobias confounds multiple sources of “threat” that are unique from (and indeed may be driven by) a dispositional threat sensitivity. So, narrowing the scope of the scale is a necessary trade-off to ensure increased accessibility and utility. However, we recognize that others may consider that trade-off to have created potential implications for the scale’s scope. Some may wish to use the 10-item version from step 3, and we would not outright discourage that if acceptable internal reliability is present. It could be argued that a more comprehensive battery of objects and situations would allow researchers to capture a broader swath of potential sources of physical threat. So, although we maintain that a single factor is sufficient to capture the narrow operationalization of threat sensitivity employed herein, we encourage other to continue expanding the scale by offering supplemental factors capturing unique types of threat.

## Conclusion

Across four studies, we developed and validated an easy-to-administer 5-item self-report measure of dispositional threat sensitivity. This measure can be applied to index individual differences both as a predictor, moderator, covariate, and outcome. The development of this measure has and will contribute to the many fields of psychological research that posit a role for threat in driving outcomes and bring clarity to the role of dispositional threat sensitivity in disposing people to certain judgments and behaviors.

### Supplementary Information

Supplementary Information.

## Data Availability

All data are available upon request to the first author.
