# Supplementary material for: The threat sensitivity scale: A brief self-report measure of dispositional sensitivity toward perceiving threats to physical harm
Source: Sci Rep. 2024 May 17;14:11304. doi: 10.1038/s41598-024-61476-7 (PMC11101408; doi:10.1038/s41598-024-61476-7)
Supplement: Supplementary file 1 — Supplementary Information. [file 41598_2024_61476_MOESM1_ESM.docx]

Supplemental Materials for:

The Threat Sensitivity Scale: A Brief Self-Report Measure of Dispositional

Sensitivity toward Perceiving Threats to Physical Harm

David S. March*, Connor Hasty, & Vincenzo Olivett

Florida State University

**Belief in a Dangerous World (BDW) Scale**

Instructions: Please rate the following statement on how much you believe they are true. 1 means you don't believe it is true at all, 7 means you believe it is very true. (1 = Not true at all, 7 = Very true).

It seems that every year there are fewer and fewer truly respectable people, and more and more persons with no morals at all who threaten everyone else.

Although it may appear that things are constantly getting more dangerous and chaotic, it really is not so.

Every era has its problems, and a person's chances of living a safe, untroubled life are better today than ever before.

If our society keeps degenerating the way it has been lately, it's liable to collapse like a rotten log and everything will be in chaos.

Our society is not full of immoral and degenerate people who prey on decent people. News reports of such cases are grossly exaggerating and misleading.

1. The "end" is not near. People who think that earthquakes, wars and famines mean God might be about to destroy the world are being foolish.
2. There are many dangerous people in our society who will attack someone out of pure meanness, for no reason at all.
3. Despite what one hears about "crime in the street," there probably is not any more now than there ever has been.
4. Any day now, chaos and anarchy could erupt around us. All the signs are pointing to it.
5. If a person takes a few sensible precautions, nothing bad will happen to him. We do not live in a dangerous world.
6. Every day, as our society becomes more lawless, a person's chances of being robbed, assaulted, and even murdered go up and up.
7. Things are getting so bad, even a decent law-abiding person who takes sensible precautions can still become a victim of violence and crime.
8. Our country is not falling apart or rotting from within.

**The Disgust Propensity and Sensitivity Scale - Revised (DPSS-R)**

Instructions: Please read each statement and think how often it is true for you. (1 = Never, 2 = Rarely, 3 = Sometimes, 4 = Often, 5 = Always)

1. I avoid disgusting things.
2. When I feel disgusted, I worry that I might pass out.
3. It scares me when I feel nauseous.
4. I feel repulsed.
5. Disgusting things make my stomach turn.
6. I screw up my face in disgust.
7. When I notice that I feel nauseous, I worry about vomiting.
8. I experience disgust.
9. It scares me when I feel faint.
10. I find some things disgusting.
11. It embarrasses me when I feel disgusted.
12. I think feeling disgust is bad for me.

**Behavioral Inhibition/Activation Scales (BIS/BAS)**

Instructions: Each item of this questionnaire is a statement that a person may either agree with or disagree with. For each item, indicate how much you agree or disagree with what the item says. Please respond to all the items; do not leave any blank. Choose only one response to each statement. Please be as accurate and honest as you can be. Respond to each item as if it were the only item. That is, don't worry about being "consistent" in your responses. Choose from the following four response options:

1 = very true for me

2 = somewhat true for me

3 = somewhat false for me

4 = very false for me

BIS:

1. If I think something unpleasant is going to happen I usually get pretty "worked up."
2. I worry about making mistakes.
3. Criticism or scolding hurts me quite a bit.
4. I feel pretty worried or upset when I think or know somebody is angry at me.
5. Even if something bad is about to happen to me, I rarely experience fear or nervousness.
6. I feel worried when I think I have done poorly at something.
7. I have very few fears compared to my friends.

BAS-Reward:

1. When I get something I want, I feel excited and energized.
2. When I'm doing well at something, I love to keep at it.
3. When good things happen to me, it affects me strongly.
4. It would excite me to win a contest.
5. When I see an opportunity for something I like, I get excited right away.

**Curiosity About Morbid Events Scale (CAMES)**

1. I like to watch sports like prize-fighting or ice hockey that sometimes get a bit violent. (T)
2. Most horror movies are fairly amusing. (T)
3. Pornography is a healthy outlet for frustrated sexual needs. (T)
4. I would like to see an autopsy being performed. (T)
5. Most pornographic (X-rated) movies are fairly amusing. (T)
6. I enjoy being mildly frightened by horror movies. (T)
7. If I could travel back in time to ancient Rome I would be curious enough to visit the coliseums to watch gladiators fight each other and wild animals to the death. (T)
8. I like to look at nude pictures of attractive persons of the opposite sex. (T)
9. Under no circumstances would I like to watch other persons having sexual relations. (F)
10. Television news focuses too much on the violent effects of accidents, war, and crime. (F)
11. When I see a serious auto accident on the road und it is apparent that there is no need for further help. I still slow down in order to see what happened. (T)
12. Most horror movies are disgusting. (F)
13. I enjoy reading descriptions of sex in novels or stories. (T)
14. I am curious about crime and therefore usually read the detailed news accounts about murders and other violent crimes. (T)
15. I would like to see a bull-fight. (T)
16. I am not interested in watching car races because the drivers are sometimes killed or seriously injured in them. (F)
17. People who like to look at or read pornography must be sick. (F)
18. Under no circumstances would I like to see a person being killed. (F)
19. I enjoy being mildly aroused by sexy movies. (T)
20. I am not particularly interested in looking at nude pictures of attractive persons of the opposite sex. (F)
21. I think I would like to witness an execution. (T)
22. It does not bother me to see extreme violence portrayed in movies or television. (T)
23. There is altogether too much explicit sex shown in movies. (F)
24. Television news should show us the results of war and crime, no matter how gory, so we do not have any illusions about these topics. (T)
25. I do not generally read detailed news accounts of murders and other violent crimes. (F)
26. I think I would like to watch other persons having sexual relations. (T)
27. I would not want to look at a dead person. (F)
28. I would not like to watch a major surgical operation being performed. (F)

**Modern Morbid Curiosity Scale (MMCS)**

Directions: Rate how much attention or interest you would exhibit if you were to see each of the following objects or situations. There are no right or wrong answers, just answer honestly and with your gut reaction. (1 = I would pay no attention, 2 = I would pay a little attention, 3 = I would pay an average amount of attention, 4 = I would pay a fair amount of attention, 5 = I would pay a lot of attention).

1. A car accident
2. A siren
3. Caution/police tape
4. A loud noise
5. A run-down building
6. Someone in public yelling
7. Screaming in the distance
8. Someone running in a non-running environment
9. An ambulance
10. A police report

**Ten-Item Personality Inventory (TIPI)**

Instructions: Here are a number of personality traits that may or may not apply to you. Please write a number next to each statement to indicate the extent to which you agree or disagree with that statement. You should rate the extent to which the pair of traits applies to you, even if one characteristic applies more strongly than the other.

1 = Disagree strongly

2 = Disagree moderately

3 = Disagree a little

4 = Neither agree nor disagree

5 = Agree a little

6 = Agree moderately

7 = Agree strongly

I see myself as:

1. Extraverted, enthusiastic. (E)
2. Critical, quarrelsome. (A, reversed)
3. Dependable, self-disciplined. (C)
4. Anxious, easily upset. (ES, reversed)
5. Open to new experiences, complex. (OE)
6. Reserved, quiet. (E, reversed)
7. Sympathetic, warm. (A)
8. Disorganized, careless. (C, reversed)
9. Calm, emotionally stable. (ES)
10. Conventional, uncreative. (OE, reversed)
